# MALDI Imaging Mass Spectrometry (MALDI-IMS)—Application of Spatial Proteomics for Ovarian Cancer Classification and Diagnosis

**DOI:** 10.3390/ijms12010773

**Published:** 2011-01-21

**Authors:** Johan O. R. Gustafsson, Martin K. Oehler, Andrew Ruszkiewicz, Shaun R. McColl, Peter Hoffmann

**Affiliations:** 1 Adelaide Proteomics Centre, School of Molecular and Biomedical Science, The University of Adelaide, SA 5005, Adelaide, Australia; E-Mails: ove.gustafsson@adelaide.edu.au (J.O.R.G.); shaun.mccoll@adelaide.edu.au (S.R.M.); 2 Robinson Institute, Research Centre for Reproductive Health, School of Paediatrics and Reproductive Health, The University of Adelaide, SA 5005, Adelaide, Australia; E-Mail: martin.oehler@adelaide.edu.au; 3 Surgical Pathology, SA Pathology, Adelaide SA 5000, Australia; E-Mail: andrew.ruszkiewicz@health.sa.gov.au

**Keywords:** MALDI, imaging, mass spectrometry, ovarian cancer, grading, biomarker

## Abstract

MALDI imaging mass spectrometry (MALDI-IMS) allows acquisition of mass data for metabolites, lipids, peptides and proteins directly from tissue sections. IMS is typically performed either as a multiple spot profiling experiment to generate tissue specific mass profiles, or a high resolution imaging experiment where relative spatial abundance for potentially hundreds of analytes across virtually any tissue section can be measured. Crucially, imaging can be achieved without prior knowledge of tissue composition and without the use of antibodies. In effect MALDI-IMS allows generation of molecular data which complement and expand upon the information provided by histology including immuno-histochemistry, making its application valuable to both cancer biomarker research and diagnostics. The current state of MALDI-IMS, key biological applications to ovarian cancer research and practical considerations for analysis of peptides and proteins on ovarian tissue are presented in this review.

## 1. Epidemiology of Ovarian Cancer

In 2010, an estimated 21 880 new cases of ovarian cancer will be diagnosed in the USA [1]. http://www.cancer.org/research/cancerfactsfigures/cancerfactsfigures/cancer-facts-and-figures-2010.

With a projected 13 850 deaths from this disease in 2010, ovarian cancer has the highest mortality rate of all gynaecological malignancies. The high mortality from ovarian cancer is due to the majority of patients (64%, see Table 1) being diagnosed with advanced (International Federation of Gynecology and Obstetrics (FIGO) stage III + IV) disease, which has a maximum 5-year survival of only 30% [2]. In contrast, the 5-year survival for patients with organ-confined FIGO stage I ovarian cancer exceeds 90% and a large number of these patients are cured. Thus, early detection is the key to increased survival in ovarian cancer.

## 2. Early Detection of Ovarian Cancer

While other gynaecological cancers can be diagnosed at an early stage due to effective screening (e.g., PAP smear in the case of cervical cancer) or symptoms (e.g., bleeding in the case of endometrial cancer), neither specific early disease symptoms or an early detection test exist for ovarian cancer. Presently, diagnosis involves a combination of physical examination, followed by trans-vaginal ultrasound, measurement of serum levels of the glycoprotein CA-125 and exploratory surgery if a suspicious ovarian lesion has been identified [1]. CA-125, when combined with ultrasound, has a positive predictive value (PPV) of only 35.1% for primary EOCs [2]. This low PPV indicates that two out of every three patients will be over diagnosed and undergo unnecessary and potentially harmful invasive procedures. Novel biomarkers are therefore required to improve ovarian cancer detection.

Single markers, such as CA125, are unlikely to provide the sensitivity and specificity required for ovarian cancer screening [3]. The focus has thus shifted to panels of biomarkers, which for the moment are additional diagnostic tools, not screening options [4,5]. Further improvements to these panels require not only discovery of new biomarkers, but also validation of existing biomarker candidates. Moreover, the large numbers of newly identified potential biomarkers have to be validated individually in a large cohort of patients, which is currently impractical. The use of serum or plasma for many biomarker discovery projects also complicates the process of biomarker discovery, as serum has a high protein complexity, large dynamic range of protein concentration (10^12^) [6] and contains non-specific acute phase proteins. A more promising approach is therefore the direct analysis of the cancer tissue, as it should have the highest concentration of disease specific markers [7] and a smaller dynamic range of protein concentration (10^6^) [8]. Thus, by focusing on tissue identified candidates it should be possible to compile a smaller subset of biomarkers with a higher specificity which can be validated *in situ* by immuno-histochemistry (IHC) and subsequently in large patient cohorts by established methods like enzyme linked immuno-sorbent assays (ELISA). These biomarkers could then be used in novel high specificity panels for early diagnosis of ovarian cancer.

## 3. Molecular Classification of Ovarian Carcinomas

The absence of reliable biomarkers is not the only issue with respect to ovarian cancer diagnosis. Following histologic confirmation of ovarian disease, treatment is assigned based upon stage [1]. Ovarian cancer staging is currently defined by the FIGO classification system for tumour dissemination into extra-ovarian sites (see Table 1), which correlates well with patient five year survival (see SEER http://seer.cancer.gov/statfacts/html/ovary.html)[9,10]. However, grade is an additional important prognostic parameter [11]. Grade, as determined by light microscopy describes morphological characteristics of tumour tissue including percentage of solid growth, architecture, nuclear features and mitotic activity (see Table 2). [12]. These characteristics are subjective and their reproducibility may be suboptimal [12]. Moreover, contention exists as to which grading systems most accurately reflect ovarian tumour differentiation status and therefore optimal treatment [12,13].

Based on recent advances in the understanding of the molecular biology of ovarian cancer it is now believed that the major ovarian cancer subtypes can be separated (see Table 2) into type I (low grade) or type II (high grade) based upon differential gene and/or protein expression [13–16]. These two-tiered molecular systems of ovarian cancer grading provide an avenue for defining cancer differentiation state in absolute terms. As such, molecular grading systems need to be developed to a point where they can complement routine histo-pathological examination of ovarian cancer tissue. Importantly, this also needs to be achieved on a similar time scale to histology, in this case one to two hours.

Thus, to improve EOC management and outcome for patients, both discovery of novel, effective biomarkers and development of a new molecular grading/classification system are required.

## 4. Application of Proteomics to Ovarian Cancer

Although gene expression is useful for distinguishing ovarian tumour subtypes [14], it does not always correlate with protein translation [17,18], nor can levels of post translational modification (PTM) be directly inferred from genetic analyses [19]. However, both protein expression level and PTM state have drastic effects on cellular function/dysfunction and as a result it is more meaningful to analyse the disease-related proteins and peptides. Generating protein profiles with sufficient molecular features is impossible with IHC, as it is limited to a maximum of three to four antibodies at a time and, crucially, depends on antibody quality. Proteomics, however, allows analysis of hundreds to thousands of peptide and protein features in biological samples [20], in many cases without the need for antibodies.

The term “proteomics” was coined to describe the quantitative analysis of the proteome, which represents all proteins expressed in a given cell, tissue (e.g., cancer) or biological fluid (e.g., serum) at a given point in time or under the effects of a defined biological stimulus [21]. High analytical sensitivity is achieved in proteomics because complex protein mixtures are fractionated following tissue or cell lysis (disruption), followed by further purification or direct analysis by mass spectrometry (MS) [22,23]. These methods allow for identification of thousands of proteins from a single cell lysate. For example, two separate studies from 2006 [24] and 2008 [19] demonstrated profiling of ovarian cancer subtypes using liquid chromatography (LC) separation followed by MS (LC-MS). The 2008 study showed that early and late stage endometroid ovarian carcinoma MS profiles can be distinguished using a clustering analysis, which separates profiles based on feature similarity; in this case similar protein masses [19]. Importantly, the 2008 publication also combined profiling MS data for serous and clear cell tumours from the 2006 study [24] to show that the three subtypes grouped separately in a principal component analysis (PCA). These studies are significant as they indicate that “classical” proteomics can generate molecular fingerprints of disease. However, there are two issues for implementing proteomics in this manner. Firstly, tissue disruption for analysis removes spatial proteome information, which is critical for clinical application, especially in heterogeneous carcinomas where different structural elements will express a unique proteome with subsequent unique cellular function. A common method for addressing this problem is laser capture micro-dissection (LCM) [7], which can isolate specific cell populations for analysis. However, similar to many proteomics methods, including liquid phase separation, LCM is time consuming. The second issue is thus that a proteomic method is required that can be implemented in the same time frame as classical histology (*i.e.*, one to two hours).

## 5. Tissue Analysis by Mass Spectrometry

Direct tissue section analysis utilising an MS instrument removes the need for disruption and the subsequent loss of spatial proteome information. This approach also provides an avenue for molecular classification/grading because tissue sections can be prepared and analysed rapidly (1–3 hours) using standardised protocols. Importantly, tissue specific biomarkers can be visualised and subsequently identified using “classical” proteomics methods such as LC-MS. For easy reference, the advantages and disadvantages of methods for analysing tissues (histology, IHC and proteomics) are summarised in Table 3.

MS measurement of molecules directly from tissue was first described in 1997 [25]. MS instruments measure the mass to charge ratio (*m/z*) of gaseous ions, in this case peptide or protein ions. Mass is of value because it indicates composition, which, for example, can be used to identify proteins of interest by their component peptides. To generate ions directly from tissue, either secondary ion MS (SIMS) or matrix-assisted laser desorption/ionisation (MALDI) instruments are utilised.

SIMS utilises the impact of an ion beam (e.g., Ar^+^ or Ga^+^) to induce a localised gain in kinetic energy on the tissue surface. Once a sufficient energy level is reached secondary ions (e.g., peptides) are ejected from the tissue for mass measurement [26]. In practice, SIMS causes surface fragmentation and as a result limits measurement to metabolites, lipids and small peptides (<1000 Da) [26].

MALDI represents a more suitable ionisation method for direct application to tissue. Preparation for MALDI requires a tissue section to be coated with a low molecular weight organic molecule, called the “matrix”. The most common matrix compounds, 2,5-dihydroxybenoic acid (DHB), α-cyano-4-hydroxycinnamic acid (CHCA), and sinapinic acid (SA) are listed in Table 4 along with example modifications made to the matrix composition, their full chemical names and bio-molecule specificity. Several of these matrix combinations have been applied to ovarian tissue including DHB/3-AP [27], CHCA [28], CHCA/ANI [28], SA [28,29], SA/3-AP [30] and SA/HFIP [30,31]. Most matrixes are dissolved in a 50–60% acidified organic solvent solution, which extracts lipids, peptides and proteins from the tissue prior to evaporation, allowing the matrix to crystallise. The end result is a field of sample-matrix co-crystals on the tissue surface.

**Figure 1:**
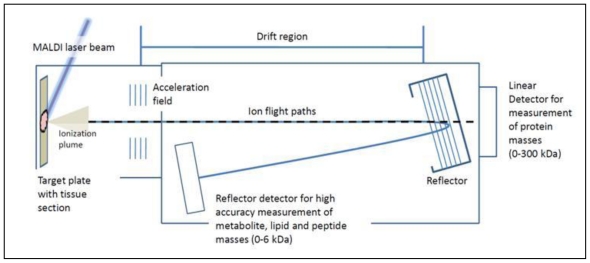
Basic principles of matrix-assisted laser desorption/ionisation (MALDI) time-of-flight (TOF) mass spectrometry. Following ionisation, sample ions are accelerated into an electric field free “drift” region. The larger the ion the less energy it will gain during acceleration and as a result it will travel slower than smaller ions. This is the basis of time of flight separation. Time from laser ionisation to detection at the opposite end of the drift region is used to determine mass to charge ratio (*m/z*) for masses between 0–300 kDa. High mass accuracy is achieved using a reflector field that focuses ions from 0–6 kDa onto a secondary detector.

MALDI is achieved by directing a laser beam at the co-crystals. The matrix absorbs the bulk of incident laser energy, causing an explosive transition from solid crystal to a gaseous plume, during which ionisation of the sample occurs (see Figure 1) [32–35]. MALDI is suited to bio-molecule analysis because it is a “*soft”* ionisation process, in that the matrix is the energy absorber, minimising protein/peptide fragmentation. MALDI ion sources are typically coupled to time-of-flight (TOF) mass analysers. Ions from the MALDI process are accelerated into the TOF tube, which is an electric field free flight region. The kinetic energy gained during acceleration decreases with increasing mass and as such heavier ions will fly slower and therefore have a longer time-of-flight. This is the basis of TOF mass analysis. When an ion hits an attached detector, the time from laser ionisation to detection is used to derive *m/z* (see Figure 1). The end result is a plot of *m/z* against intensity (ion counts); commonly referred to as an MS spectrum. The preference of most groups for MALDI-TOF instruments is due to their sensitivity (femto to attomolar range under ideal conditions), ease of use and achievable mass range of MALDI-TOF, which reaches from small molecules (100 Da) to large proteins (>300 kDa), allowing measurement of metabolites, lipids, peptides and proteins on the same instrument.

The key advantages of MALDI-TOF MS application directly to tissue are thus that:

- Several bio-molecule classes from different mass ranges can be measured, including drugs [36], lipids [27], peptides [37,38] and proteins [31,39,40].
- Several hundred molecular features can be measured in a single experiment (see Figure 3a–c).
- No preliminary knowledge about tissue composition is required.
- No antibodies are required.

## 6. Methods for *in Situ* MALDI-TOF Analysis of Ovarian Cancer Tissue

**Figure 2:**
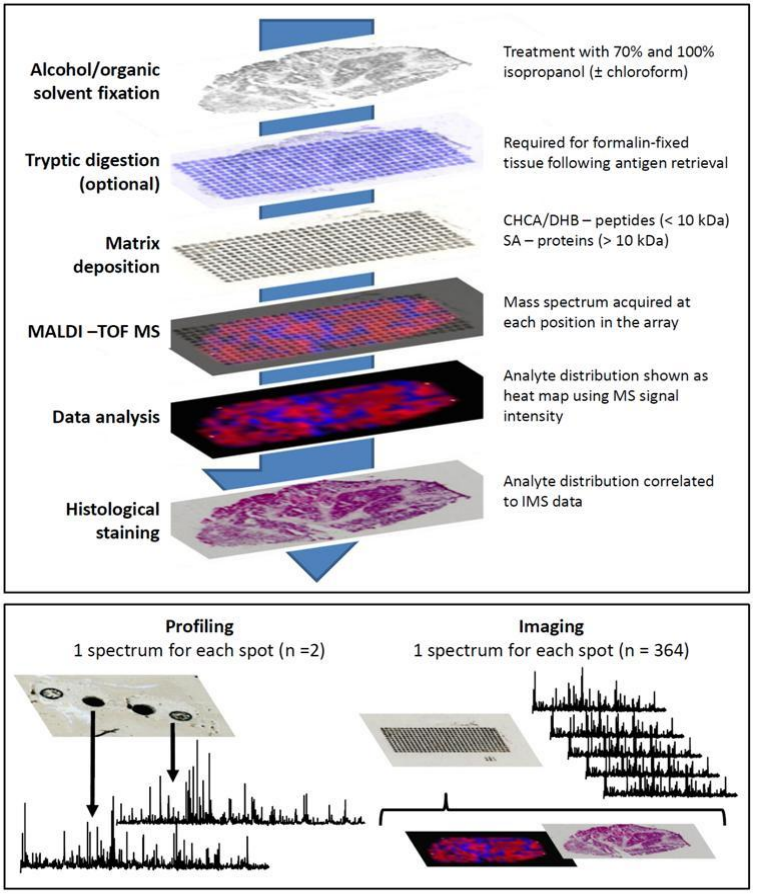
The top panel shows a typical workflow for IMS on ovarian tissue. Note the optional tryptic digest (absolute requirement for formalin-fixed paraffin embedded (FFPE) tissue). Antigen retrieval can also be used to partially hydrolyse formalin-induced protein cross-links. The bottom panel shows the two analysis workflows possible for an IMS experiment, profiling and imaging.

**Figure 3:**
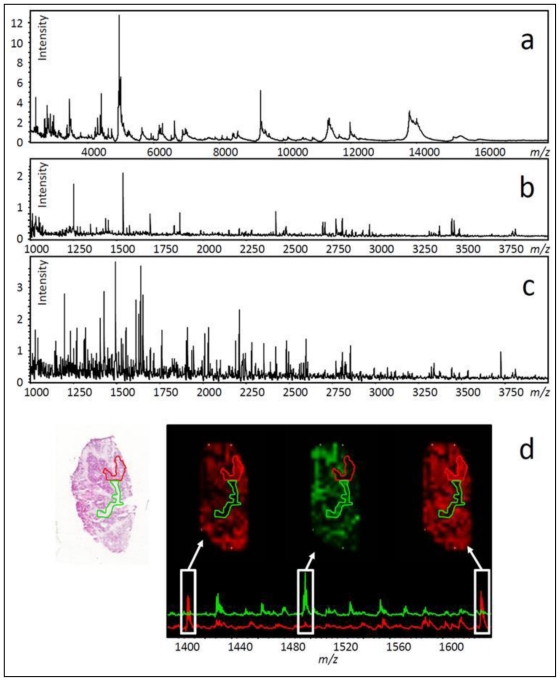
Data from printed arrays on stage IIIC ovarian epithelial carcinoma. The spectra in a-d represent the sum of all spectra for small protein (**a**–matrix only + peptide/ small protein mass range), endogenous peptide (**b**–matrix only + peptide mass range) and tryptic peptide (**c**–trypsin digestion + matrix with peptide mass range) analysis using a MALDI-TOF/TOF MS instrument. Twenty mg/mL DHB in 50% methanol and 0.2% trifluoroacetic acid was used as a matrix. Trypsin was used at 40 ng/μL in a 5 mM NH_4_HCO_3_ and 12% acetonitrile buffer at pH ~8.5. Panel d shows two morphologically different areas on a H&E stained section (green/red outlines), previously analysed by tryptic peptide IMS. Three ion intensity maps with associated spectra for the green and red areas show differential peptide distribution.

An outline of the methodology for *in situ* MS analysis of ovarian tissue is shown in Figure 2. Sectioned tissue (2–10 μm thick) is mounted directly onto chilled conductive glass slides (indium tin oxide coated) or metallic targets (e.g., gold coated target). The mounted sections are dried (15–45 min) before fixation with a graded alcohol series (70% and 100% v/v ethanol/isopropanol) or organic solvents such as chloroform or acetone [41–44]. This fixation has been shown to increase the quality of MS signals, most likely as a result of physiological salt and lipid removal, both of which interfere with matrix crystallisation and subsequent quality of MS data [42,43]. For ovarian tissue, washes with 70% and 100% isopropanol are sufficient to generate rich small protein (Figure 3a) and endogenous peptide (Figure 3b) MS signals directly from carcinoma sections. However, chloroform can also be used quite successfully as a stand-alone wash prior to analysis [30]. As described previously, to allow MALDI measurement, the tissue is coated with a matrix solution. The method of matrix coating is important as it affects the type (single spectra or multiple) and quality (MS sensitivity) of data obtained. These methods are discussed further as they pertain to the two types of *in situ* MALDI-TOF MS experiment, profiling and imaging MS (IMS).

## 7. Profiling Cancer Tissues Using MALDI-TOF MS

Typically, *in situ* MS methods are split into two types of workflows, these being profiling MS or IMS (see Figure 2, bottom panel). The profiling MS approach uses manual or automated deposition of matrix at discrete locations on a tissue section. MS spectra are then acquired from these positions and compared. If tissue MS profiles are known from previous analyses, the same tissue type can be identified in future studies based on this profile, a process similar to the MALDI Biotyper platform used for identification of micro-organisms [45]. Because of the novelty of IMS research, there are few publications dealing exclusively with ovarian cancer. However, successful examples of molecular classification/grading have been published for non-small cell lung cancer (NSCLC) [46] and soft tissue sarcomas (STS) [47]. In the lung cancer study, 100 nL of sinapinic acid (SA, see Table 4) matrix was manually spotted onto 42 NSCLC tumours (>70% tumour cellularity) and 8 normal lung sections. MS data was acquired in a *m/z* range of 2–25 kDa on a MALDI-TOF MS system and 82 peaks specific to the cancerous tissue were selected for development of a class prediction model. The training model generated was applied to a validation set of 32 tumour and 5 normal lung sections. Based on the MS data all 32 tumours of the validation set were classified correctly as tumour or normal. Moreover, segregation of these mass signals based on tumour subtype, in this case 14 adenocarcinoma, 15 squamous cell carcinoma and 5 large cell carcinoma, allowed for 100% separation of adenocarcinomas and squamous cell as well as squamous and large cell tumours. Only one large cell tumour was mis-classified as adenocarcinoma in the study [46]. The STS study was able to distinguish low and high grade STS using MALDI IMS profiling. Drops of SA matrix (200 nL) were applied directly to areas showing cellular proliferation following MALDI-compatible cresyl violet staining [47,48]. It was determined that calgizzarin (S100 A11), calcyclin, macrophage inhibitory factor and calgranulin were potentially diagnostic for high grade STS, with key extracellular proteins such as myosin being down-regulated in both low and high grade tumours as compared to control muscle tissue. These findings were confirmed using IHC against sections of tumour and control tissue [47].

In addition, pilot experiments have been published for grading follicular lymphoma [49], detection of pre-invasive bronchial lesions [50] and prostate cancer [51,52], classification of meningiomas [53], and generation of prognostic information for gliomas [54]. These pilot studies have shown the utility of characterising disease via direct MS tissue section analysis to gain diagnostic [47] and prognostic data [54].

The most comprehensive study to date analysing EOC [30] profiled tissue from 19 ovarian tumours (10 benign, 6 carcinoma and 3 borderline). Three mass ranges were examined, combining data from typical IMS peptide (CHCA matrix, see Table 4) and small protein (SA matrix) analysis with a novel method for extracting high molecular weight proteins using SA dissolved in 1,1,1,3,3,3-Hexafluoro-2- propanol (HFIP). Using stage III and IV tumours, as compared to benign tissue, it was possible to profile masses matching those of cancerous marker proteins previously identified in EOC, including tetranectin (17.7 kDa) and urokinase plasminogen activator (36.9 kDa) [30]. Figure 4 highlights the differences between tissues as a plot of *m/z* against sample number as well as a PCA loadings plot showing separation of the benign, carcinoma and borderline tissues.

## 8. Profiling *vs.* Imaging

Sample preparation for profiling involves deposition of larger (100–500 nL) drops of matrix onto discrete positions of the tissue section. In contrast, IMS methods require nebulisation of a homogeneous layer of matrix or deposition of a rectangular array of smaller droplets (0.1–0.2 nL) onto tissue sections (Figure 2, bottom panel). The benefit of IMS is that instead of documenting profiles for singular locations, the relative abundance (based on MS signal intensity) of hundreds of protein or peptide ions is mapped across an entire tissue section at a centre to centre acquisition distance of 250 μm or smaller. This is achieved by combining all spectra, acquired from a matrix array coating a single tissue section, into a sum spectrum. Mass filters are applied to the sum spectrum, which subsequently mines data from the individual spectra in the data set, presenting the normalised intensity of individual mass ranges as a 2-D heat map (see Figure 3d). It is this heat map, otherwise known as an ion intensity map, which allows visualisation of peptide and protein distribution across a tissue section. It is thus possible to document changing molecular profiles as tissue composition changes, a process which can be likened to molecular histology [55].

## 9. Software for Data Analysis

Several software platforms are currently available, which generate ion intensity maps from spatially referenced IMS data. A selection of IMS software platforms that were available as of 2008 were listed in Jardin-Mathe *et al.* (2008) [56] along with important features. Most vendors offer IMS software packages for their MS instruments, including Shimadzu Biotechnology (Intensity Mapping software), AB-SCIEX (TissueView software), Bruker Daltonics (flexImaging software), Waters (conversion tool to use BioMAP) and Thermo Fisher Scientific (ImageQuest software).

Additional freeware programs available include Novartis BioMap, data cube explorer (AMOLF, Amsterdam, Netherlands), fxSpectViewer (CEA, Saclay, France), Mirion (Justus Liebig University, Giessen, Germany) and the MALDI imaging team imaging computing system (MITICS, Lille, France) [56]. Of the mentioned software, only BioMap and data cube explorer are readily available by download. Data cube explorer, for example, uses a universal IMS file format “imzML” to avoid problems with file compatibility for newly developed software. ImzML, is based on the proteomics standard mzML [57] and is currently being proposed as a global IMS standard because it maintains the spatial coordinate system of IMS data in a universally recognisable format; in this case a smaller file for meta data and a larger binary file for the MS data (see http://www.maldi-msi.org/ and Römpp *et al.* (2011) [58]). The widespread use of imzML would allow IMS researchers to directly access publicly available datasets, compare data sets to their own, and compile analysis scripts to accompany published data. Both BioMap and data cube explorer are available for download from http://www.maldi-msi.org/, along with tutorials on usage.

## 10. Automated Sample Preparation for Imaging Cancer Tissues

IMS matrix deposition can be achieved using manual deposition of dry matrix powder via a sieve or sublimation, nebulising instruments such as handheld air brushes [55] and the Bruker Daltonics ImagePrep station [39], or printers such as the Labcyte Portrait [59] and Shimadzu Chemical Inkjet Printer (ChIP-1000) [60]. Table 5 summarises important features, advantages and disadvantages of the four most common matrix deposition methods. Similar to matrix choice, deposition method can also be bio-molecule specific. For example, dry deposition or sublimation of matrix leads to poor incorporation of larger molecules such as peptides and proteins into matrix crystals because there is no extracting solvent. As a result this type of deposition is typically employed for IMS of metabolites and lipids, which have a higher ionisation efficiency. The air brush and ImagePrep station are more efficient in terms of sample incorporation into the matrix crystals and are suitable for all bio-molecule types (optimisation of methods may be necessary). However, only experienced users should attempt air brush deposition of matrix, as volume, flow and subsequently reproducibility are difficult to control (see Table 5). Greater control is possible for nebulisation using the ImagePrep, where matrix solution is gravity fed onto a porous metal film, which is vibrated by current flow through an attached piezoelectric sheet. As a result, the matrix is vaporized (nebulised) and settles as a dense mist onto the tissue section. Matrix deposition in the ImagePrep is controlled by measurement of light scatter, which increases with greater crystal density. These nebulised preparations generate a homogeneous matrix field where the spatial acquisition resolution is usually limited to 20–50 μm for the homogeneous matrixes (CHCA and SA, see Table 4), although higher resolution work has been reported for manual spray preparations [55]. A steady loss of MS sensitivity (*i.e.*, ion count intensity) is experienced as resolution is increased, as a result of the smaller area and therefore smaller amount of sample being analysed. Importantly, the push button functionality of the ImagePrep and its standardised methods make it a viable candidate for clinical application.

Printed IMS arrays are, in effect, whole tissue profiling experiments generated by repeated deposition of picolitre volumes of matrix in a rectangular grid (see Figure 2). Deposition of matrix in this manner limits users to a maximum acquisition of resolution determined by the droplet size on the tissue which can vary from 150-250 μm, centre to centre, depending on the quality of the preparation.

The ChIP-1000, for example, uses a pressure manifold to maintain solution in a reservoir mounted on top of a 55 μm printing nozzle. Droplets ranging from 100–200 pL are ejected using force generated by current flow through a piezo electric material. The principal down side to the ChIP-1000 is the nozzle itself, which can clog with crystallised matrix. In terms of printing, DHB is the most stable ChIP-1000 matrix. Because DHB is water soluble, and water is not as volatile as organic solvents, printing can be performed for hours without direct supervision. However, several solid ionic matrixes (e.g., CHCA with molar excess of aniline, see Table 4) have been developed that increase print stability by reducing the rate of CHCA and SA crystallisation [61]. The gold standard for printed arrays is a nozzle free system such as the Labcyte Portrait printer, which focuses sound waves at the surface of a matrix solution. Turbulence at the surface ejects droplets (of similar size to the ChIP-1000) vertically onto the tissue section which is suspended, face down, above the solution tray [59]. The high cost of this instrument and methods to overcome matrix clogging issues on the ChIP-1000 have unfortunately prevented widespread application of the Portrait.

As already discussed, there is a balance between sensitivity and spatial resolution. Because the volume of matrix deposited is greater for printers than nebulising instruments, sample extraction efficiency is also greater, leading to improved MS sensitivity. However, for the purposes of a grading approach there is typically no reason to implement the highest resolution nebulised IMS methods, with most studies settling for a 100–200 μm spatial resolution [39]. Moreover, deposition of printed arrays or singular spots onto a tumour section, whether guided by histology or independent of it, is more than sufficient to generate MS profiles for grading and biomarker detection [28,30,62].

## 11. Peptide Imaging Provides Data Complementary to Protein Imaging

Despite advances such as HFIP solvent for improved protein extraction, for the moment, IMS is limited to masses below 70 kDa [31], preventing ready detection of higher molecular weight proteins such as cell surface receptors. Moreover, MS sensitivity decreases dramatically as protein mass increases. Consequently, only the very highest abundance high molecular weight proteins will be observed. To circumvent these issues, it is possible to perform *in situ* proteolytic digests by deposition of enzymes such as trypsin. The digested tissue is coated with matrix (homogeneous layer or printed array) and MALDI-TOF MS acquisition is performed in the peptide mass range (0–6 kDa, see Figure 1). The resulting peptide MS spectra (Figure 3c) are vastly more complex than the protein level (Figure 3a). However, digest methods allow (i) higher molecular weight proteins to be analysed via their component peptides, (ii) fragmentation of highly abundant peptides directly from tissue to gain sequence information [63] and (iii) direct extraction from the tissue and identification using LC-MS methods, which are well established in most proteomics facilities [64].

## 12. Using Histology to Guide Imaging Mass Spectrometry

After MS acquisition is complete, the matrix crystals can be removed using ethanol to allow histological staining and assessment by a pathologist. MS compatible stains such as cresyl violet can also be used prior to IMS to guide analyses [47,48]. Importantly, good correlation between MS ion intensity maps and anatomical structures has been demonstrated previously for various tissues including neuroendocrine [65], breast [39] and ovarian cancer (see Figure 3d) [64]. This correlation shows the value of IMS as a complement to histology.

The ability to correlate histology and IMS data was exploited recently for investigation of the changing molecular profile of tumour interfaces. Upon analysis of the tumour boundaries of renal cell carcinoma, the definition of “normal” surrounding tissue has been called into question, with demonstration of potential tumour associated protein changes appearing well past the histological tumour margin [66]. In a separate study on serous ovarian carcinomas, IMS was used to show that the tumour interface zone contains a unique set of MS detectable masses as compared to tumour and surrounding normal tissue [29]. IMS can thus generate molecular data which is unique and novel to that provided by morphology alone.

## 13. Ovarian Cancer Biomarker Discovery Using Imaging Mass Spectrometry

In developing the IMS technology, preliminary IMS biomarker discovery projects for ovarian cancer have been reported by the same group that presented profiling work on ovarian carcinomas (see Section 7). Putative biomarkers of ovarian cancer were detected using printed IMS arrays and subsequently identified using LC-MS/MS of digested cancerous tissue [30]. These included 11S proteasome activator complex Reg Alpha fragment [28], oviductin (mucin-9) [30] and orosomucoid [30], the roles of which are described briefly here. Reg-Alpha, or PA28, is an antigen processing protein, an increased expression of which may allow presentation of self peptides on tumour cells, and subsequently immune evasion [28,30]. Oviductin is a marker of oviductal epithelium and tubal differentiation marker [30,67], and finally orosomucoid is an acute phase protein previously evaluated as a marker of ovarian cancer and possible immune suppressor through action on T lymphocytes [30,68]. It is clear that relevant markers of disease can be identified. However, more work is required to determine how effectively MS profiles of such markers can distinguish the subtypes of ovarian cancer and how well these markers translate to cancer detection and screening.

## 14. Application of Tryptic Digestion to Formalin-Fixed Paraffin Embedded Ovarian Tissues

Frozen tissue represents the current gold standard for IMS, given that a freshly preserved tissue will harbor a freshly preserved proteome, which is easy to access using standard methods for both protein and tryptic peptide IMS (see Figure 3). However, the limited archival life of frozen tissue (maximum two years for proteomics applications) has forced researchers to adopt methods for accessing peptide and protein mass data in formalin-fixed paraffin embedded (FFPE) tissue; the current global standard for long term tissue preservation in medical centres and research laboratories worldwide [69–71]. However, formalin fixation induces cross-linking between multiple amino acid side chains, creating a linked protein network [72]. To access these tissues by MS, antigen retrieval (AR) [62,64] and/or *in situ* tryptic digestion [73,74] are required. While AR is not completely understood, its most likely effects are to partially hydrolyse cross-links and denature linked proteins. Typically, this is insufficient for subsequent MS acquisition of the same quality as frozen tissue, because the cross-linking is not reversed completely. Thus, AR is usually followed by tryptic digestion [62].

**Figure 5:**
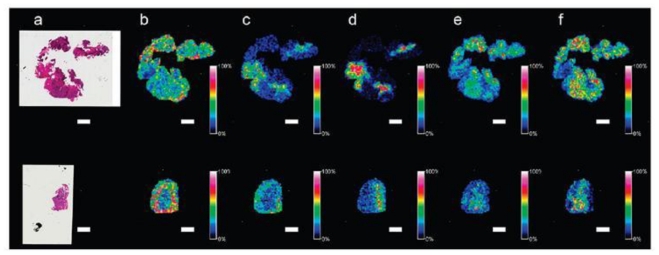
MALDI-TOF IMS of formalin-fixed paraffin-embedded (FFPE, top row) and frozen (bottom row) ovarian carcinoma. FFPE sections were treated with antigen retrieval. Frozen sections were washed using a previously described protocol. Both sections were digested with trypsin and coated with CHCA matrix using an ImagePrep station. Figure reprinted, with permission, from Gustafsson *et al.* 2010 [64]. Scale bars = 2 mm.

Several publications have so far demonstrated successful application of AR methods for IMS on various tissues [62,74,75], including FFPE ovarian cancer [64,73]. A 2007 study applying tryptic digestion alone to de-paraffinised and rehydrated sections of ovarian cancer showed that many high abundance proteins could be identified directly from tissue [73]. However, this study was a proof of principle application and as such did not demonstrate disease specific distribution of peptides generated from the tissue sections. Our own group has successfully applied citric acid antigen retrieval to ovarian cancer for tryptic peptide IMS (see Figure 5). Peptides were also extracted from *in situ* proteolytic digests and identified using liquid phase peptide separation and MS. Using this method it was possible to assign tentative identities to 48 individual peptides [64]. Because of the ability to rapidly extract and identify peptides directly from tissue using “classical” fractionation-based proteomics, the translation from peptide IMS data to peptide identification and subsequent *in situ* validation by IHC becomes less labour intensive. Furthermore, the large existing archives of FFPE ovarian tissue will allow any acquired peptide IMS data to be matched to patient history and clinical outcome.

## 15. Conclusions and Future Prospects

The successful application of MS profiling and IMS for tissue classification and grading has been demonstrated for different types of cancer. In the case of ovarian cancer, preliminary studies have isolated and identified potential tissue specific peptide and proteins masses using IMS. The current aim is continued application of *in situ* MS methods to demonstrate acquisition of ovarian cancer grade and/or subtype specific protein/peptide profiles from both frozen and archived FFPE tissues. Importantly, from further investigation, IMS derived markers could be used to track molecular changes across ovarian tumours as well as their interfaces with normal tissue to determine the importance of subsequent protein and peptide masses as tissue markers. Following selection of these specific markers, identification, as already demonstrated in several publications, can be achieved using classical proteomics at either the protein or peptide level (fractionation/identification methods such as LC-MS). Subsequent validation of these masses using IHC will ultimately indicate the suitability of masses for further development as diagnostic markers for ovarian cancer sub-type or grade and for validation in large patient cohorts as biomarkers.

## Figures and Tables

**Figure 4:**
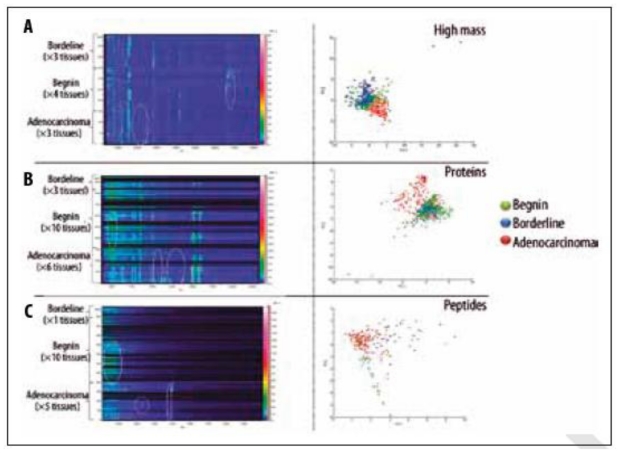
Figure from El Ayed *et al.* 2010 showing MALDI-TOF MS profiling on three classes of ovarian tissue (adenocarcinoma, borderline and benign). Plots of m/z against spectral source as well as loadings plots from principal component analysis are included for high mass proteins (**A**), small proteins (**B**) and peptides (**C**). Figure reprinted, with permission, from El Ayed *et al.* 2010 [30].

**Figure 4:**
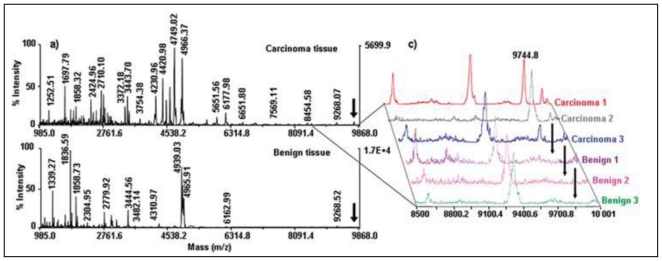
MALDI-TOF mass spectrometry profiles of ovarian carcinoma and benign tissue sections (**a**). The individual profiles of three carcinomas and three benign tumour sections are shown in (**c)**, with the mass at 9744.8 m/z highlighted as the potential biomarker 11S proteasome activator complex Reg Alpha fragment. Figure reprinted, with permission, from Lemaire *et al.* (2007) [28].

**Table 1:** FIGO ovarian cancer stages, prevalences and anatomical features.

FIGO Stage	Prevalence (%)	Anatomical features
I	25	Limited to ovaries
II	11	Pelvic extension
III	47	Abdominal extension and/or positive lymph nodes
IV	17	Distant metastases

**Table 2:** Grading systems for epithelial ovarian carcinoma: FIGO, universal three tier grading and two tier grading.

Grading system		Grade	Key features			Ref.
FIGO		1	Well differentiated	Grade based on % solid non-squamous growth, grade + 1 if nuclear atypia apparent	<5% solid growth	[10]
		2	Moderately differentiated		6–50% solid growth	
		3	Poorly differentiated		>50% solid growth	[13]
3-tier universal grading		1	Grade based on sum of individual feature scores (see right) 1 = 3–5 points 2 = 6–7 points 3 = 8–9 points	Architecture based score	Glandular = 1 point Papillary = 2 points Solid = 3 points	
		2		Nuclear pleomorphism score	Slight = 1 point Moderate = 2 points Marked = 3 points	[12]
		3		Mitotic activity score	0–9 = 1 point 10–24 = 2 points ≥25 = 3 points	
2-tier grading	Serous tumour	Low grade (type I)	Slow development	Low chromosomal instability	Gene mutation–KRAS, BRAF, ERBB2	[14]
		High grade (type II)	Rapid development	High chromosomal instability	Gene mutation–P53	
	Endomet roid tumour	Low grade	Well differentiated, no necrosis	Solid glandular architecture	Gene mutation–Wnt, PI3K/Akt	[13]
		High grade	Solid growth >50%, necrosis	Diffusely infiltrative or expansive growth, no glandular architecture	Gene mutation–TP53	[16]

**Table 3:** Comparison of different methods (histology, immuno-histochemistry (IHC) and proteomics (fractionation coupled to mass spectrometry (MS) and direct tissue MS) for peptide/protein analysis in tissue samples.

	Histology	IHC	Proteomics	
			Fractionation-MS	Direct tissue MS
**Methods**	Cellular staining	Antibody directed staining of specific proteins	Liquid phase separation (*i.e*., liquid chromatography)	Direct measurement of peptides and proteins from tissue section
**Analysis**	Tissue morphology assessment by light microscopy	Protein distribution across tissue sections	MS protein identification	MS profiles of tissue sections
			Quantitation using protein labelling	Peptide and protein intensity maps showing distribution across tissue sections
**Advantages**	Easy staining methods	Highly specific	Highly sensitive	Rapid
	Cellular microscopy resolution	Cellular microscopy resolution	Thousands of proteins analysed at a time	Spatial proteome information
	Well established	Well established	Heavily automated	Measurement of hundreds of molecular features at a time
	Clinical personnel already available	Clinical personnel already available	Highly modular workflows	No antibodies required
**Disadvantages**	Reproducibility issues	Time consuming	Time consuming	Expensive equipment
	Based on visual assessment of morphology	Labor intensive	Labor intensive	Novel technology
	Non-specific	Limited to 3–4 proteins	Removes spatial information	Requires fraction-MS based proteomics to identify peptide and protein features
	Analysis is subjective	Dependent on antibody quality	Requires specialist personnel	Analytical resolution limited to a maximum of 20–50 μm

**Table 4:** List of the three most common matrix types—2,5-dihydroxybenzoic acid (DHB), α-cyano-4-hydroxycinnamic acid (CHCA) and 3,5-dimethoxy-4-hydroxycinnamic acid (sinapinic acid, SA) as well as their documented modifications - for MALDI mass spectrometry. Suitability for measurement of bio-molecules is specified [26].

Matrix	Chemical name	Biomolecule specificity
DHB	2,5-dihydroxybenzoic acid	Lipids, peptides, <10 kDa proteins
DHB/aniline	DHB + aniline	Lipids, peptides, <10 kDa proteins
DHB/3-AP	DHB + 3-acetyl pyridine	Lipids, peptides, <10 kDa proteins
CHCA	α-cyano-4-hydroxycinnamic acid	Peptides, small proteins (<10 kDa)
CHCA/aniline	CHCA + aniline	Peptides, <10 kDa proteins
SA	3,5-dimethoxy-4-hydroxycinnamic acid	Proteins (>10 kDa)
SA/aniline	SA + aniline	Proteins (>10 kDa)
SA/3-AP	SA + 3-acetyl pyridine	Proteins (>10 kDa)
SA/HFIP	SA + 1,1,1,3,3,3-hexafluoro-2-propanol	Proteins (>30 kDa)
SA/TFE	SA + 2,2,2-trifluoroethanol	Proteins (>30 kDa)

**Table 5:** Summary of reproducibility, acquisition resolution, the advantages and the disadvantages of four different matrix deposition methods are listed here for air brushes and the ImagePrep station (matrix nebulising/spray instruments) as well as the ChIP-1000 and Labcyte Portrait (matrix printing instruments).

	Nebulising instruments		Printers	
	Air brush	ImagePrep station	ChIP-1000	Labcyte Portrait
**Reproducibility**	Poor	Good	Excellent	Excellent
**Acquisition resolution**	≥5 μm	≥20 μm	≥150 μm	≥150 μm
**Advantages**	Cheap	Automated	Automated	Automated
	High resolution MS acquisition	High resolution MS acquisition	Control over reagent volume deposited	Control over reagent volume deposited
	Good for start up imaging MS laboratories	Default methods available but methods can be modified by user	High MS sensitivity	High MS sensitivity
**Disadvantages**	Lower peptide/protein incorporation into matrix	Lower peptide/protein incorporation into matrix	Expensive	Most expensive
	Requires experienced user	Requires experienced user	Time consuming preparation	Time consuming preparation
	Manual preparation	Expensive	Lower data acquisition resolution than nebulised preparations	Lower data acquisition resolution than nebulised preparations
